# Comparing Non-Invasive Spectrophotometry to Hematology Analysis for Hemoglobin Measurements in Sickle Cell Disease Patients

**DOI:** 10.3390/jcm12247517

**Published:** 2023-12-05

**Authors:** Khaled Yassen, Nawal Omer, Fatimah Alsahaf, Fatima Al Amer, Fatimah Alhamad, Imran Alherz, Abdulaziz Bushehab, Fatma Alniniya, Maryam Alwabari

**Affiliations:** 1 Anesthesia Unit, Surgery Department, College of Medicine, King Faisal University, Hofuf 31983, Al Ahsa, Saudi Arabia; 2 Hereditary Blood Disease Center, Hofuf 36422, Al Ahsa, Saudi Arabia; gelas3@hotmail.com; 3 College of Medicine, King Faisal University, Hofuf 31983, Al Ahsa, Saudi Arabia; fatimah.sahhaf@gmail.com (F.A.); fatima.alamer595@gmail.com (F.A.A.); fatma19992013@gmail.com (F.A.); alwabari.maryam.e@gmail.com (M.A.); 4 Anesthesia Department, King Fahad Hospital, Ministry of Health, Hofuf 36441, Al Ahsa, Saudi Arabia; dr.imran.alherz@gmail.com (I.A.); majbourha@gmail.com (F.A.); 5 Nursing Services, Hereditary Blood Disease Center, Hofuf 36422, Al Ahsa, Saudi Arabia; abdulazizbushehab@gmail.com

**Keywords:** hemoglobin, monitoring, non-invasive, sickle cell disease

## Abstract

**Highlights:**

The following are some of the highlights of our study:

- The SpHb concentration was found to be higher than the lab Hb concentrations, with a positive correlation.
- SpHb measurement sensitivity and precision were lower than expected.
- Non-invasive SpHb measurement can play a role in excluding anemia in outpatient clinics.
- The PI values among the SCD patients were lower than the patients with a normal hemoglobin structure.
- SpHb measurement requires technological improvements to overcome the low PI observed in SCD patients.

**Abstract:**

Patients with sickle cell disease (SCD) require repeated blood sampling for hemoglobin (Hb) concentration measurements. The primary aim of this study was to compare non-invasive spectrophotometric hemoglobin (SpHb, g/dL) measurements to those taken via an automated hematology analyzer (Hb, g/dL) in patients with SCD visiting outpatient clinics and to investigate the correlations and agreements between both measurement techniques. Secondarily, we aimed to identify the SpHb cut-off concentration for the diagnosis of anemia and to monitor the effects of the pleth variability index (PVI, %) and perfusion index (PI) on SpHb measurements. The results gained from the examination of one hundred and fifty-eight patients indicated that the SpHb measurements overestimated the lab Hb concentrations, with a mean (SpHb-Hb) bias of 0.82 g/dL (SD 1.29). The SpHb measurements were positively correlated with the Hb measurements (Kendall’s tau correlation (τ), *n* = 158, τ = 0.68, *p* < 0.001), with an intra-class correlation (ICC) of 0.67 and a 95% CI from 0.57 to 0.74 (*p* = 0.000). The SpHb cut-off concentration to diagnose anemia was 11.4 and 11.7 g/dL for males and females, respectively. SpHb sensitivity was low for males and females at 64.4% and 57.1%; however, the specificity was higher at 90.9% and 75%, with positive predictive values (PPVs) of 95.6 and 85.7, respectively. No correlation existed between SpHb measurements and the PVI (%) in contrast with a moderate correlation with the PI (r = 0.049, *p* = 0.54, and r = 0.36, *p* < 0.001, respectively). The mean PI was low at 2.52 ± 1.7. In conclusion, the SpHb measurements were consistently higher than the lab Hb concentrations, with a positive correlation. The sensitivity and precision of the SpHb measurements were lower than expected. However, the SpHb specificity and its positive predictive values (PPVs) indicated that it is less likely for a patient with a positive SpHb test result for anemia to be non-anemic. These results will allow SpHb measurement to play a role in excluding the presence of anemia. In light of the low PI values determined, the SpHb measurements were challenging to take and, thus, require further technological improvements.

## 1. Introduction

Sickle cell disease (SCD) is a known health problem in the Kingdom of Saudi Arabia, particularly in the east, where the disease is prevalent [1,2]. SCD is a disease that demonstrates an autosomal recessive inherited pattern and results from a mutation in the beta-globin chain of the hemoglobin (Hb) structure, where valine takes the place of glutamate at position six [3,4,5]. Anemia is one of the most common complications of SCD, which is the consequence of the precipitation of abnormal hemoglobin inside red blood cells, which has deleterious effects on cell membranes and blood vessels. This ultimately leads to the destruction of red blood cells and hemolysis. Frequent venous blood sampling is mandatory to monitor hemoglobin levels and the response to therapy in SCD patients. Venous blood sampling is distressing for children as well as adults. Several non-invasive devices that require no blood sampling for the measurement of hemoglobin in the blood are now available. Hiscock and colleagues (2015) discussed the variations in accuracy and precision between these devices in their systematic review and meta-analysis [6]. Recently, Okazaki and colleagues (2022) shared their experience of the non-invasive monitoring of Hb concentrations among schoolchildren [7]. In 2023, Ali Syed and colleagues tested a wristband with optical-based sensors and succeeded at continuously and non-invasively monitoring Hb concentrations among SCD patients from the Middle East. They calculated the extent of light transmittance through the fingers of SCD patients by utilizing Beer–Lambert’s law [8,9].

Variations in accuracy between these devices are still the focus of debates [10,11,12,13,14,15], and there are a few recently published studies examining patients with SCD [8,16].

The device used in the current study is a point-of-care multi-wavelength (500 nm to 1300 nm) pulse oximetry device from Masimo Radical 7, Irvine, CA, USA, designed to non-invasively measure hemoglobin, and the Hb measured is named spectrophotometric hemoglobin (SpHb, g/dL) [9]. Szmuk et al. (2011) and Al-Khabori et al. (2021) are among the few researchers to have monitored hemoglobin among SCD patients [15,16]. Unfortunately, both studies were presented as abstracts during scientific meetings and were not published in full. Al-Khabori et al.’s study [16] is one of the few studies that includes SCD patients from the eastern area of Saudi Arabia. Of note, the Asian haplotype is more frequently present in the Middle East [17].

### Aim of this Study

The aim of this study was to compare hemoglobin concentrations (SpHb, g/dL) measured via a point-of-care multi-wavelength pulse oximetry device to hemoglobin concentrations (Hb, g/dL) measured via a hematology analyzer among a group of SCD patients. Any correlations and agreements found were also studied. Secondarily, we aimed to identify the SpHb cut-off values for identifying anemia; values lower than 13 g/dL for males and 12 g/dL for females are the values used by the World Health Organization (WHO) to define anemia [18]. Finally, we aimed to analyze the effect of the pleth variability index (PVI, %) and perfusion index (PI) on SpHb measurements.

## 2. Materials and Methods

The current diagnostic test accuracy study was approved by the Al-Ahsa Health Cluster (IRB KFHH No.: H-05-HS-065; IRB Log No.: 08-EP-2023), Hofuf City, Kingdom of Saudi Arabia. The information presented here concerns the second part of this study. The first part of this study focused only on patients with normal hemoglobin concentrations and was published recently [19]. The current research represents the second part of this study and focuses on a different population of patients, namely, patients with abnormal hemoglobin as a result of sickle cell anemia. Both parts were approved by the above-mentioned local ethics committee (IRB Log No.: 08-EP-2023).

### 2.1. Patients

This study was conducted at the Hereditary Blood Disease Center in Hofuf City, Al Ahsa, Saudi Arabia, between 26 January and 1 May 2023. All patients’ demographics and clinical laboratory data were retrieved from the patients’ records. The criterion for inclusion was as follows: all SCD patients visited outpatient clinics during the study period for regular check-ups and treatment follow-up. All included patients had to have been in a steady clinical state, adults (>18 years) from both genders, and of all phenotypes. The exclusion criteria included patients < 18 years, patients who were hemodynamically unstable, and patients in any form of crisis or in need of a blood transfusion. Refusal to consent to monitoring and sampling or patients with missing data also fell within the exclusion criteria.

### 2.2. SpHb, Pleth Variability Index (PVI, %), and Perfusion Index (PI)

Specific finger probe sensors for SpHb (g/dL), PVI, %, and PI (Masimo Radical 7, Irvine, CA, USA) were utilized. SpHb was calculated non-invasively via an optical-shielded fingertip probe with Rainbow signal extraction technology from Masimo, which emits light with multiple wavelengths (575 nm to 1100 nm), and based on the adsorption of light in blood, it calculates the hemoglobin concentration non-invasively [20,21]. 

The PVI is a continuous, non-invasive parameter that demonstrates the variability in pleth during respiratory cycles. It is a dynamic indicator of fluid responsiveness with a normal range between 9 and 13%.

The PI is the ratio of pulsatile blood flow to non-pulsatile static blood flow measured in the fingertip. The perfusion index is the strength of the pulse at the sensor. PI values range from 0.02% (very weak blood flow) to 20% (very strong blood flow). A PI value of >2% is sufficient for an accurate signal.

PI = AC/DC × 100% and PVI% = PImax − PImin/PImax × 100%

Here, AC is the absorption of infrared light by the pulsating arterial inflow and DC is the constant absorption of infrared light by the skin and other tissues. High PVI values mean higher PI variability with respiration, which means that the patient will respond to fluid intake by means of an increase in cardiac output (fluid responder).

A period of five minutes prior to taking the SpHb readings was necessary to reduce any movement interference. A PI value above 0.3 is required for reliable SpHb measurements. In cases where there was no or a poor signal, the patient was excluded.

### 2.3. Laboratory Hemoglobin Measurement

The patients’ hemoglobin concentrations were measured with a hematology analyzer called Sysmex XN 100i (Sysmex Europe SE, Norderstedt, Germany) [22,23]. Phlebotomy specialists carried out all of the necessary venous blood samplings in conjunction with the SpHb measurements.

Cyanide-free sodium lauryl sulfate (SLS) was used with the Sysmex system to lyse the red blood cells and white blood cells in the venous blood samples. This SLS hydrophilic particle later binds to the heme and forms a colored SLS–HGB compound. The degree of absorption of the monochromatic light through the SLS–HGB complex indicates the hemoglobin concentration [24].

### 2.4. Measurement Time and Site

SpHb and venous lab Hb sample values were recorded at the same time during the outpatient visit. Oxygen saturation percentages (%) were also monitored, as they are reported to affect SpHb accuracy, according to Gomma et al. [25].

### 2.5. Statistical Analysis

Data were logged with the SPSS program for statistical analysis (ver. 21) [26]. Completion of the Kolmogorov–Smirnov test of normality revealed significance in terms of the distribution of some of the variables [27]. Independent non-normally distributed data were compared with the Mann–Whitney U test [28], and correlations and agreements were tested with the non-parametric Kendall’s tau correlation (τ) [29], intra-class correlation (ICC) [30], and Bland–Altman assessment [31,32].

The sample size was calculated according to a previous study by Okazaki et al. [7]. A minimum sample size of 59 patients was adequate for this agreement study with a significance level of 5% (α error accepted = 0.05) and statistical power (1 − β) of 80%.

## 3. Results

Overall, 160 patients were enrolled in the study between 26 January 2023 and 1 May 2023, but only 158 patients were included in the final study. Two patients had poor PI signals (<0.3) and were subsequently excluded. In the study population, there were 96 (61.6) males and 58 (38.4) females. These results represent part two of our study, with this part focusing on patients suffering from sickle cell disease characterized by an abnormal hemoglobin structure. The first part of this study focused on patients with a normal hemoglobin structure and was recently published [19].

Table 1 shows the laboratory data, which include the mean ± SD of SpHb (g/dL), Hb (g/dL), PVI (%), and PI. 

**Figure 1:**
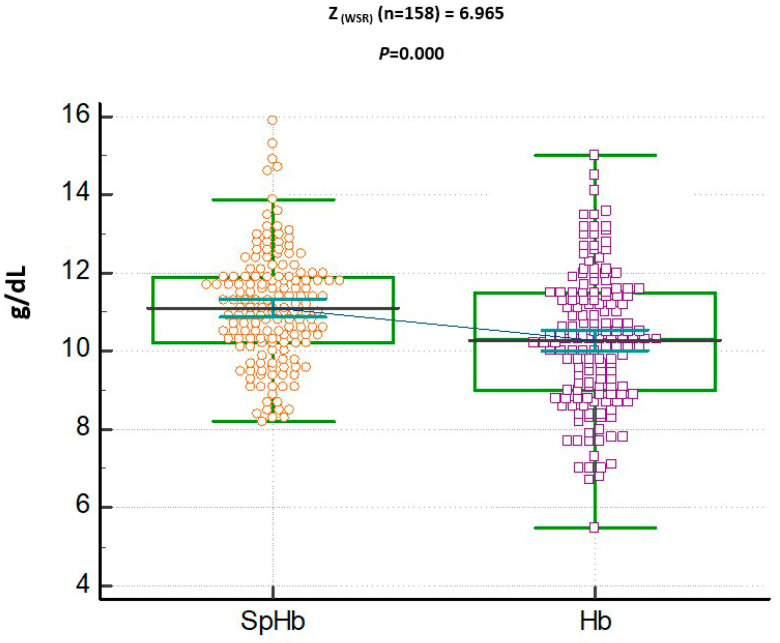
Dot plots with connecting lines for the median and 95% confidence interval (CI) of both spectrophotometric hemoglobin (SpHb, g/dL) and laboratory hemoglobin (Hb, g/dL). SpHb overestimated the Hb concentration values. WSR: Wilcoxon signed rank test performed.

In Table 1, the mean (SD) SpHb concentration (11.10 + 1.43 g/dL) was higher than the mean (SD) of the lab Hb (10.27 + 1.73 g/dL; *p* < 0.001; Figure 1).

The mean PVI was 16.73 ± 7.69%, and the mean ± SD perfusion index (PI) was low at 2.52 ± 1.7, as shown in Table 1. This low PI value demonstrates the reduced quantitative measure of perfusion in this group of patients suffering from SCD. A weak correlation existed between the PVI (%) and SpHb (g/dL) (r = 0.049, *p* = 0.54), while a low to moderate correlation was observed between the PI and SpHb (r = 0.36, *p* < 0.001). Table 1 also includes the detailed hemoglobin electrophoresis of the studied patients. The mean ± SD of Hb F was 16.77 ± 7.63 g/dL, with no difference between patients on hydroxy urea medication and those who were not, as shown in Table 2.

### SpHb and Hb Relationship

**Figure 2:**
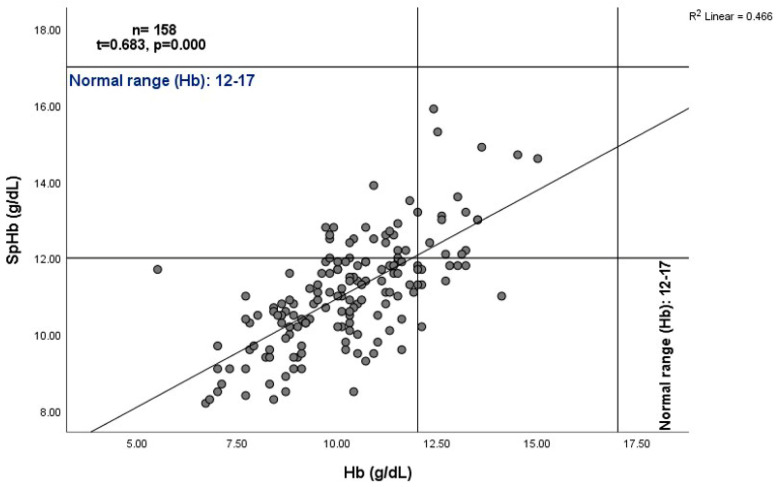
Simple scatter graph with a regression (best fit) line showing a moderate and positive correlation between spectrophotometric hemoglobin (SpHb, g/dL) and laboratory hemoglobin (Hb, g/dL).

**Figure 3:**
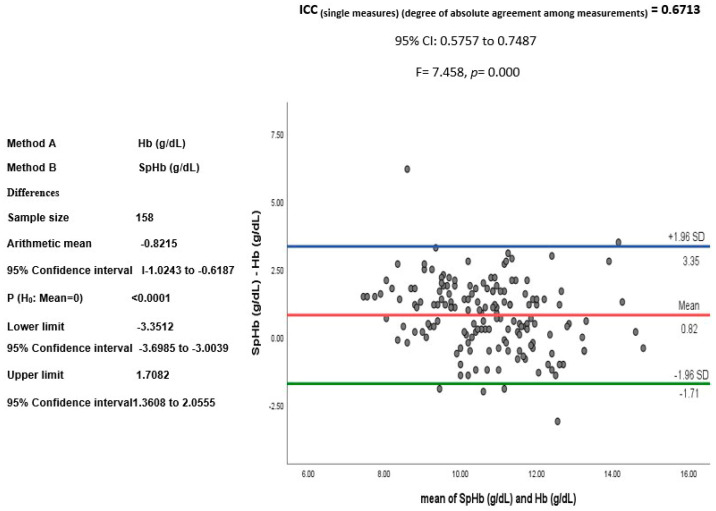
Agreement between spectrophotometric hemoglobin (SpHb, g/dL) and laboratory hemoglobin (Hb, g/dL) demonstrated by the intra-class correlation (ICC).

The blood concentrations of SpHb (11.10 + 1.43 g/dL) were overestimated in comparison to those of the lab Hb (10.27 + 1.73 g/dL; *p* < 0.001), with a positive correlation (*n* = 158, τ = 0.68, *p*< 0.001), as shown in Figure 1 and Figure 2. A good degree of reliability was also demonstrated between Hb (g/dL) and SpHb (g/dL) (*n* = 158 readings), with a bias (SpHb-Hb) of 0.82 g/dL (SD 1.29) and significant intra-class correlation (*p* = 0.000; Figure 3).

**Figure 4:**
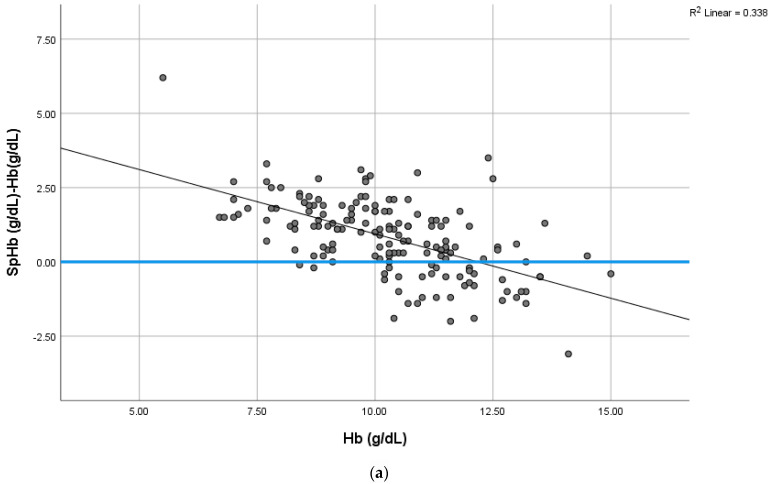

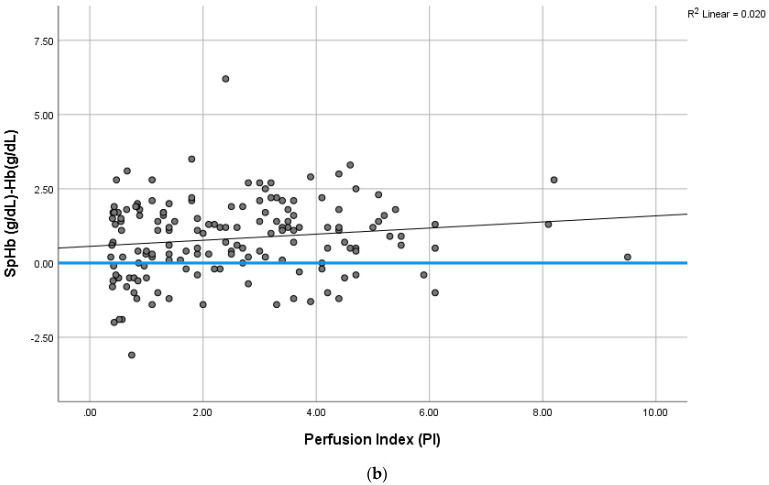
(**a**) The mean difference between SpHb and Hb (g/dL) varies from positive to negative, depending on the Hb concentration (g/dL). There is a negative difference (underestimate) at high Hb and a positive difference (overestimate) at low Hb concentrations. The bias is inversely correlated with higher Hb. This trend is illustrated by a non-parametric smoothed regression line. (**b**) The relationship between bias (SpHb–tHb) and the perfusion index (PI) is also shown. The trend is illustrated by a non-parametric smoothed regression line.

The bias (SpHb-Hb, g/dL) was inversely correlated with the Hb concentrations (Figure 4a). The relationship between this bias and the PI was insignificant, as shown in Figure 4b.

**Figure 5:**
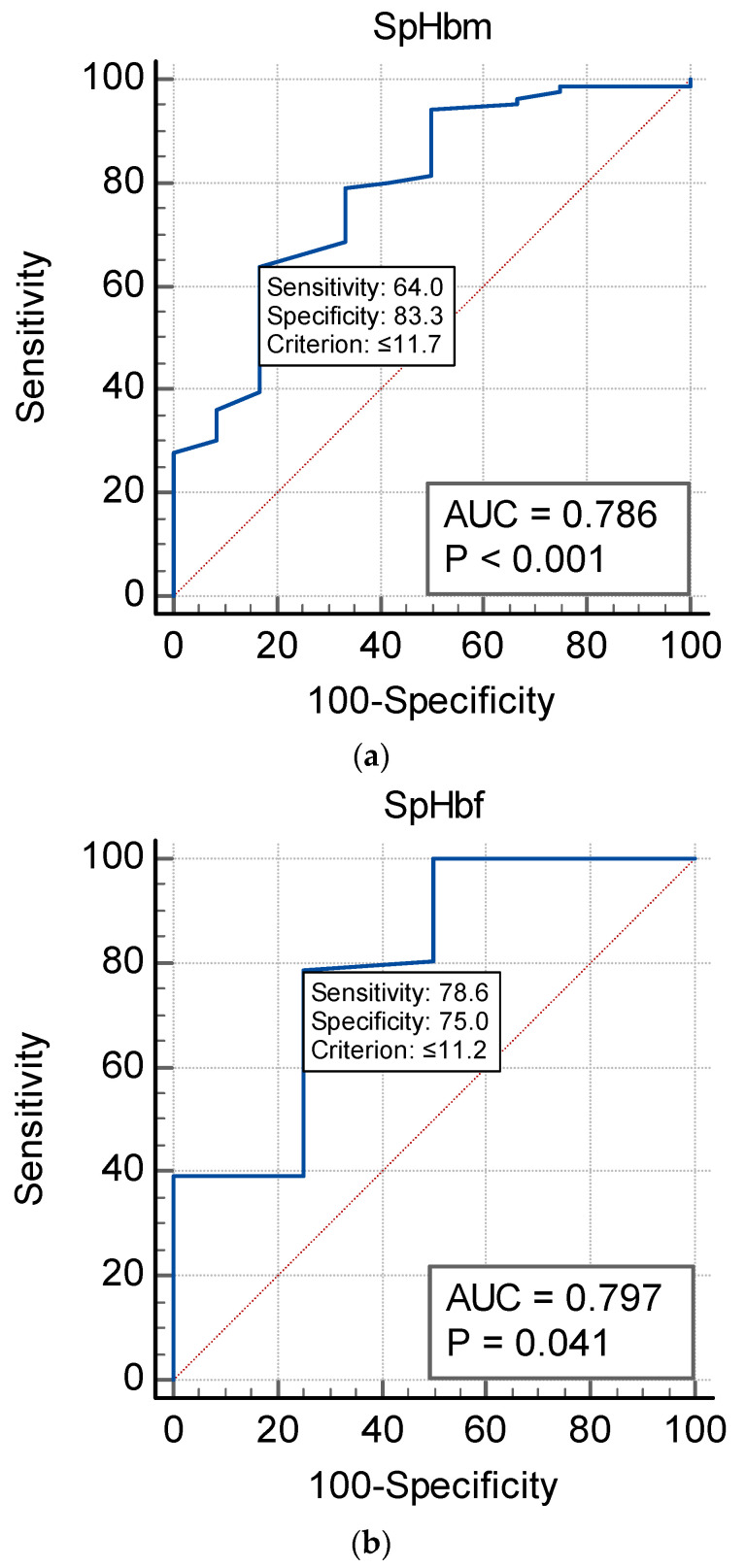

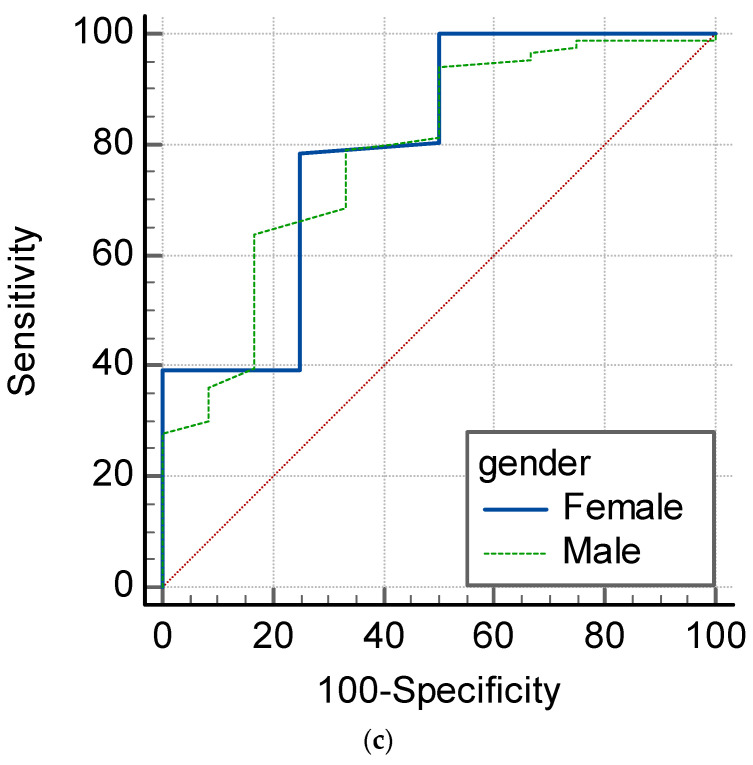
(**a**). Males’ non-invasive hemoglobin (SpHb) ROC graph demonstrating the ability of SpHb < 11.3 g/dL to diagnose anemia with a sensitivity of 83.3% and a specificity of 81.0%. Prevalence of anemia among males = 32.3%. (**b**). Females’ non-invasive hemoglobin (SpHb) ROC graph demonstrating the ability of SpHb < 10.2 g/dL to diagnose anemia with a sensitivity of 62.9% and a specificity of 91.3%. The prevalence of anemia among females = 60.3%. (**c**). Comparison of independent ROC curves for both females and males in the ROC graphs (*p* = 0.61).

The ability of SpHb to detect anemia according to the WHO definition is presented in Table 3 and Figure 5a–c. SpHb was able to identify anemia but at low sensitivity values of 64.4% and 57.1% for males and females, respectively. Specificity was higher at 90.9% and 75%, respectively. Anemia was present in 93.33% of males and 88.78% of females with SCD. 

## 4. Discussion

The current study is among the few published studies to investigate the accuracy of SpHb measurements in a group of patients with SCD and an abnormal hemoglobin structure. In the results section, the SpHb concentrations were consistently higher than the standard lab Hb concentrations; this was similar to the findings reported by Al-Khabori et al.’s study [16], which could be attributed to slow capillary blood flow in patients with SCD. The sensitivity values of the SpHb in our current study were 64.4% and 57.1% for males and females, respectively, which were lower than expected. This is in contrast to the SpHb specificity values, which were 90.9% and 75% for males and females, respectively. In a study by Young et al. performed with Masimo Pronto devices (2021) among patients with a normal hemoglobin structure, the researchers reported values for sensitivity of 45.7% and specificity of 85.3% [33]. Their results were close to the results of our current study, despite the difference in hemoglobin structure between both studies and the characteristics of the included population. In Young et al.’s study, the study population comprised healthy refugee volunteers, while in our study, the participants were patients suffering from SCD. 

Both high SpHb specificity and PPV will allow SpHb to play a role in excluding anemia. SpHb can be utilized as an initial anemia screening test, as it is less likely, based on these findings, for a patient with a positive SpHb test for anemia to be free from anemia.

Another significant finding in this specific group of SCD patients was the low PI values reported. The SpHb behavior examined through the use of infrared technology in SCD patients is seldom discussed in the literature. It is possible that these low PI values, which indicate reduced peripheral capillary circulation, can reduce light absorption through SpHb measurement devices. As such, this aspect requires further in-depth research.

### 4.1. Failure to Measure SpHb

It was possible to take non-invasive spectrophotometric hemoglobin measurements in 158 out of the 160 patients, and the recorded failure rate was only 1.25%. The failure rate was even higher in other studies conducted among patients with normal hemoglobin structure such as Czempik et al.’s cohort study (2022) (2%), Frasca et al.’s prospective study (2011) (5%), and Hornedo-González et al.’s study (8.19%) [34,35,36]. The current study is one of the few, if not the only one, to report the percentage of SpHb failure among patients with SCD. Only one registered nurse performed the SpHb monitoring measurements in this study; this could explain the low failure percentage compared to the other studies.

### 4.2. SpHb-Hb Relationship Variations

In contrast with patients with a normal hemoglobin structure, the bias, which is the difference between SpHb and Hb, was positive on this occasion at +0.82 g/dL (SD 1.29). This is similar to the positive bias reported by Al-Khabori et al., whose study was conducted among 98 SCD patients [16]. Few studies in the literature have looked into SpHb behavior among SCD patients, such as previous study and Szmuk et al.’s study. Szmuk et al.’s study was performed among children younger than 18 years of age, and the results were only presented during the American Society of Anesthesiologists annual meeting in 2011 and, unfortunately, have not been published in full-text format as of yet. The bias (SpHb-Hb) in Szmuk et al.’s study (*n* = 88) was also found to be positive in contrast with the negative bias values reported by other studies conducted among patients with normally structured Hb and was in agreement with our findings [15].

Miller W. et al. reported their experience of treating two children with SCD at the Southwestern and Children’s Medical Center, Dallas, USA [37]. They also came to the conclusion that a good correlation existed between SpHb and Hb invasively measured by a Hemocue and/or Coulter counter. They found that the non-invasive SpHb technique helped to reduce stress among children with SCD. Unfortunately, the results of the study by Miller W. et al. have also not been published in full.

Other studies in the literature among patients with a normal hemoglobin structure have reported variation in bias with values ranging between 1 and 2 g/dL above or below the standard. In Frasca et al.’s study, the bias value was 0.0 [35], and in Gayat et al.’s study, the bias value was found to be 0.56 g.L^−1^ [38]. Various conditions have been reported in the literature that can affect bias, but hemodilution is not one of them, as reported by Macknet et al. in 2020 [39]. 

The bias in our study was affected when the hemoglobin concentrations were at their extremes (high or low). In other words, the bias becomes positive at low Hb concentrations and negative at high Hb concentrations, as shown in Figure 4a. This was similar to the results reported by Gayat et al., but in their study, a Pronto-7 monitor (version 2.1.9, Masimo, Irvine, CA, USA) was used to examine patients admitted to the emergency unit [38]. The mean SpHb values in the current study overestimated the Hb concentrations derived from the complete blood count, as in Vos et al.’s RCT study (2012) and Miller et al.’s study [40,41].

The SpHb sensitivity varies between different studies. In Khalafallah et al.’s RCT study (2015) and Wittenmeier et al.’s prospective study (2021), both groups reported adequate SpHb and adequate sensitivity [10,12] in contrast with Honnef G et al.’s study (2022), an observational study, which reported lower SpHb sensitivity, similar to our findings [11]. 

### 4.3. SpHb as an Anemia Screening Monitor

In Western countries, anemia is mainly due to aging, chronic illness, and oncological diseases [18]. Our current study included patients from the Al Ahsa region, eastern Saudi Arabia, in which abnormal hemoglobin and associated anemia exist in a considerable percentage of the region. In our study population, anemia was present in 93.33% of males and 88.78% of females presenting with SCD. These results indicate that a significant proportion of the patients visiting the hereditary blood disease center suffered from anemia. The low sensitivity of SpHb presented in the statistical analysis is not acceptable for both males and females suffering from SCD, and this indicates the low precision of SpHb. However, both the specificity values and the PPV support the use of SpHb to rule out anemia for both sexes. 

Bıcılıoğlu et al. conducted a study in 2022 among patients with thalassemia and noted the same agreement between SpHb and Hb measurements. They reported their experience with SpHb as a guide for transfusion and for anemia screening among this specific group of hemoglobinopathic patients [42]. Their study is among the few research projects that have looked into the beneficial role of SpHb as a screening tool for anemia. 

Szmuk et al. utilized SpHb as a routine anemia screening tool for children with SCD (2011) [15], and it was found to be able to identify anemia in 86.78% and 93.33% of female and male SCD patients, respectively. 

In Khalafallah et al.’s study among patients with normally structured hemoglobin [15], the sensitivity to detect true anemia (SpHb < 12 g/dL) was significantly higher compared to patients with SCD, as in our study (75% vs. 57.1% in females and 93% vs. 64.4% in males). Hornedo-González et al. also suggested that SpHb has the ability to aid in ruling out the presence of anemia [36].

### 4.4. The Effect of a Low PI on SpHb

The Masimo Rainbow Radical 7 monitor utilizes the spectrophotometry principle to measure hemoglobin concentrations in the blood; of note, the device’s signals are known to be affected by peripheral vascular diseases. Wolf et al. noted abnormal readings in relation to oxyhemoglobin and total hemoglobin concentration in patients with peripheral arterial occlusive diseases [43]. SCD can lead to a reduction in capillary peripheral blood flow from entrapped rigid red blood cells and an associated increase in the sympathetic tone, which slows down the peripheral microcirculation and, hence, lowers the PI readings [44,45]. This was evident in the current study in which the recorded values of the PI were low, with a mean ± SD at 2.52 ± 1.7. This PI value is lower than that reported by other studies among patients with a normal hemoglobin structure [10,19]. In Alwabari et al.’s study, the PI values were higher among patients with a normal hemoglobin structure and normal peripheral circulation. Alwabari et al.’s study was performed by the same research team as in the current study. Extremely low PI values (<1.4) are not recommended for measurement by the manufacturers of Radical 7, as they can affect the accuracy of the measured SpHb. Miller et al.’s study (2012) succeeded in demonstrating an increase in peripheral perfusion with a digital nerve block, which subsequently increased the low PI values observed [46].

The mean ± SD value of the PI reading in our current study was 2.52 ± 1.7, but a proportion of the SCD patients had PI values below 1.4, as demonstrated in Figure 4b. This could explain the reduced sensitivity and precision of the device among this group of patients with SCD. 

The relationship between bias (SpHb–Hb) and the PI, as noted in the results of our current study, was insignificant. Park et al. also presented similar insignificant relationships between SpHb and the PI [47]. They also noticed that PI values tend to increase after general anesthesia induction, with a significant improvement in the SpHb-Hb bias from −2.8 to −0.7 observed. This improvement could be due to the induced peripheral vasodilatation and improvement in microcirculation, which are associated with the administration of general anesthesia and a reduction in sympathetic tone. Significant fluid shifts can also lead to low perfusion index values [48]. In our study, we noticed that the SCD patients’ fingers were cold and that they could benefit from the optical shield being warmed up; this could be the focus of a future study project to improve peripheral circulation. An Australian study by Khalafallah et al. (2015) noticed that patients with poor peripheral circulation, such as those suffering from Raynaud’s disease or other diseases, demonstrate low peripheral circulation and, hence, low PI values, which significantly affect the readings of SpHb [10]. 

### 4.5. Limitations

This study included only one non-invasive device and its applied technology, which limits the results and conclusions gained from this specific device. The lack of published literature for this specific group of patients with sickle cell disease restricted the discussion and limited the comparison of our results to other studies and devices. This study was designed to monitor clinically stable patients scheduled for elective clinical visits, with no data from patients in SCD crisis. This warrants future studies to focus on SCD patients admitted to the emergency department and requiring a blood transfusion. Variations in clinical status can affect the results produced.

Variations in the patients’ clinical co-existing diseases, which were not taken into consideration, may have influenced the device’s performance.

## 5. Conclusions

SpHb was consistently higher than lab Hb concentrations, with a bias of 0.82 g/dL (SD 1.29) and a positive correlation. The SpHb measurements’ sensitivity and precision were lower than expected. However, the SpHb specificity values and the positive predictive values (PPVs) indicated that it is less likely for a patient with a positive SpHb test for anemia to be free from it, and this allows for non-invasive SpHb measurement to play a role in excluding the presence of anemia in outpatient clinics. However, the PI values among the SCD patients were lower than the published PI values among patients with a normal hemoglobin structure. SpHb behavior requires further technological improvements to overcome these low PI values that exist among SCD patients. More studies including larger populations of SCD patients are required. 

## Figures and Tables

**Table 1:** The spectrophotometric hemoglobin (SpHb, g/dL), laboratory hemoglobin (Hb, g/dL), pleth variability index (PVI, %), perfusion index (PI) and Hemoglobin electrophoresis results of studied patients.

	Median	Mean	SD
SpHb (g/dL)	11	11.10	1.44
Hb (g/dL)	10.3	10.28	1.73
PVI (%)	15	16.74	7.70
PI	2.25	2.53	1.80
Oxygen saturation (%)	98	98.81	1.15
Hb A (%)	0.00	1.21	5.65
Hb A2 (%)	3.10	4.61	15.85
Hb S (%)	79.25	78.16	9.80
Hb F (%)	16.20	16.77	7.63

**Table 2:** Effect of medication (hydroxy urea).

Hemoglobin Type	Medications	*n*			*p*-Value
A	No	43	Mean	1.76	0.462
			Median	0	
			Std. Deviation	5.47	
	Yes	111	Mean	1.01	
			Median	0	
			Std. Deviation	5.75	
A2	No	43	Mean	3.21	0.497
			Median	3.00	
			Std. Deviation	1.06	
	Yes	114	Mean	5.20	
			Median	3.20	
			Std. Deviation	18.89	
S	No	43	Mean	76.28	0.145
			Median	77.60	
			Std. Deviation	13.51	
	Yes	114	Mean	78.74	
			Median	79.30	
			Std. Deviation	8.07	
F	No	43	Mean	17.27	0.612
			Median	17.10	
			Std. Deviation	9.17	
	Yes	114	Mean	16.66	
			Median	16.30	
			Std. Deviation	7.10	

**Table 3:** CBC: complete blood count; Hb: hemoglobin concentration as measured by complete blood count; SpHb: non-invasive hemoglobin concentration cut-off point; CBC: complete blood count; NPV: negative predictive value; PPV: positive predictive value; AUC: area under the curve; WHO definition for anemia: World Health Organization criteria for anemia (Hb < 12 g/dL in females (F) and Hb < 13.0 g/dL in males (M)).

Hb (g/dL)	SpHb	Sens	Spec	NPV	PPV	AUC
WHO (F) Hb < 12	11.4	57.1	75	22.2	85.7	0.60
WHO (M) Hb < 13	11.7	64.4	90.9	10.5	95.6	0.83
CBC (F + M) Hb < 12	11.2	78.6	75	21.1	91.9	0.80
CBC (F + M) Hb < 13	11.7	64	83.3	7.9	93.5	0.79

## Data Availability

Data are available from the authors upon reasonable request.
